# Chemical Compounds and Biologic Activities: A Review of *Cedrela* Genus

**DOI:** 10.3390/molecules25225401

**Published:** 2020-11-18

**Authors:** Thalya Soares R. Nogueira, Michel de S. Passos, Lara Pessanha S. Nascimento, Mayara Barreto de S. Arantes, Noemi O. Monteiro, Samyra Imad da S. Boeno, Almir de Carvalho Junior, Otoniel de A. Azevedo, Wagner da S. Terra, Milena Gonçalves C. Vieira, Raimundo Braz-Filho, Ivo J. Curcino Vieira

**Affiliations:** 1 Laboratório de Ciências Químicas, Centro de Ciências e Tecnologia, Universidade Estadual do Norte Fluminense Darcy Ribeiro, Campos dos Goytacazes, Rio de Janeiro 20000-000, Brazil; michelpassos19@gmail.com (M.d.S.P.); larasoares27@outlook.com (L.P.S.N.); mayara.azous@yahoo.com.br (M.B.d.S.A.); noemimonteiro77@gmail.com (N.O.M.); smr.imad@gmail.com (S.I.d.S.B.); braz@uenf.br (R.B.-F.); curcino@uenf.br (I.J.C.V.); 2 Instituto Federal de Santa Catarina, *Campus* Criciúma, Criciúma 88800-000, Brazil; almir.ribeiro@ifsc.edu.br; 3 Centro Universitário São Camilo, *Campus* I, Rua São Camilo de Léllis 01, Cachoeiro de Itapemirim, Espírito Santo 29304-910, Brazil; otoazevedo@gmail.com; 4 Instituto Federal de Educação, Ciência e Tecnologia Fluminense, *Campus* Campos Centro, Campos dos Goytacazes, Rio de Janeiro 28030-130, Brazil; wagnerdasilvaterra@gmail.com (W.d.S.T.); milena.uff@gmail.com (M.G.C.V.); 5 Departamento de Química Orgânica, Instituto de Química, Universidade Federal Rural do Rio de Janeiro, Seropédica, Rio de Janeiro 20000-000, Brazil

**Keywords:** Meliaceae, chemical profile, *Cedrela* genus, limonoids, biological activity

## Abstract

The genus *Cedrela* P. Browne, which belongs to the Meliaceae family, has eighteen species. Trees of this genus are of economic interest due to wood quality, as well as being the focus of studies because of relevant biologic activities as in other Meliaceae species. These activities are mainly related to limonoids, a characteristic class of compounds in this family. Therefore, the aim of this review is to perform a survey of the citations in the literature on the *Cedrela* genus species. Articles were found on quantitative and qualitative phytochemical studies of the *Cedrela* species, revealing the chemical compounds identified, such as aliphatics acid and alcohol, flavonoids, tocopherol, monoterpenes, sesquiterpenes, triterpenes, cycloartanes, steroids, and limonoids. Although some activities were tested, the majority of studies focused on the insecticidal, antifeedant, or insect growth inhibitor activities of this genus. Nonetheless, the most promising activities were related to their antimalarial and antitripanocidal effects, although further investigations are still needed.

## 1. Introduction

**Figure 1:**
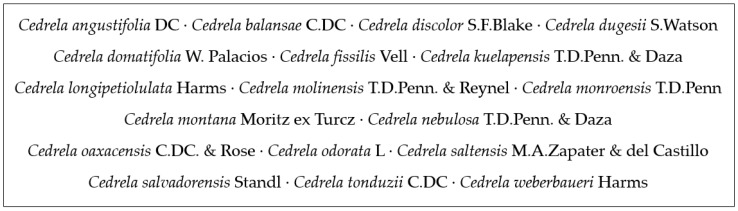
Accepted names of 18 *Cedrela* species. Data from The Plant List site (http://www.theplantlist.org/tpl1.1/search?q=cedrela) and Palacios et al. [3].

The genus *Cedrela* P. Browne contains 18 species (Figure 1) and is one of 53 genera of trees and shrubs from the Meliaceae family. These species are distributed from northern Mexico to the northwest part of Argentina [1,2,3].

Meliaceae trees are known as an excellent hardwood, prized for their wood quality, besides having considerable economic value, for example, cedar (*Cedrela*) and mahogany (*Swietenia* spp., *Khaya* spp.) [4,5]. As a result, plants of this genus have experienced overexploitation, leading to three species being added to the Official National List of Flora Species Endangered by Decree No. 443 of the Brazilian Ministry of the Environment on 17 December 2014 [6]. In this decree, *Cedrela fissilis* Vell. and *Cedrela odorata* L. were considered vulnerable species (VU), and *C. angustifolia* DC. an endangered species (EN). Furthermore, these species were also added to the International Union for Conservation of Nature (IUCN) Red List of threatened species. Therefore, several recent studies, mainly in biotechnology, have focused on sustainable production and the use of *Cedrela* in forest cultivation. Moreover, there are recommendations for the broadening of national and international legislation to protect *Cedrela* species [7].

Knowledge of the chemical compounds and, mainly, limonoids present in *Cedrela* is important to differentiate *Cedrela* and *Toona* (Endl.) M. Roem. species, considering that both genera are part of a chemotaxonomic discussion, based on the inclusion of these genera in the Cedreleae tribe within Cedreloideae by Harms [8], and in the Cedreleae tribe under Swietenioidae subfamily by Pennignton and Styles [9].

According to some authors, the affiliation of *Toona* to Swietenioideae is rather problematic, precisely because in terms of secondary metabolites, mexicanolide type limonoids, characteristic to Swietenioideae, are not present in *Toona* [10,11,12]. Thus, it could not be included in this subfamily with *Cedrela* genus, while another affirmation is that morphological aspects justify the junction of these genera by Harms [11].

Although De Leo et al. [13] present a review of terpenoids of *Cedrela* and *Toona*, this review focus is the chemical composition of only *Cedrela* species, besides current the structural classification of the limonoids isolated until now and carry out a brief discussion on the difference between *Cedrela* and *Toona* at limonoids point of view.

As reported by Tan and Luo [14], Meliaceae species attract considerable attention from the chemistry and biology research community because of possible biological activities, mainly related to the presence of characteristic limonoid compounds of this family.

Limonoids are a group of modified triterpenes, also known as tetranortriterpenoids. This class of compounds has highly oxygenated structures, derived from a precursor with a 4,4,8-trimethyl-17-furanylsteroid skeleton that is biosynthesized by the acetate-mevalonate pathway [15]. These oxidative processes and methyl group rearrangement contribute to structural diversity. It is worth mentioning that limonoids can also be found in Rutaceae, Cneoraceae, Ptaeroxylaceae, and Simaroubaceae families; however, they are especially abundant and structurally diversified in the Meliaceae family [16]

Overall, the following biological activities are described for the Meliaceae family, which are reported for the genus *Cedrela* [17]: antiviral, anthelmintic, anti-rheumatic, anti-cancer, and anti-inflammatory action, and antifeedant and insecticidal effects [4,14].

The aims of this review were to carry out a survey of the citations reported in the literature to date about species of the genus *Cedrela*, as well as to provide a foundation for further studies and the development of medicinal agents from this plant. Data were compiled from 1960 up to June 2020, focusing on all isolated compounds and also considering biological activities. 

## 2. Chemical Compounds

Since 1960, more than 200 chemical constituents have been isolated from or identified in the genus *Cedrela*, including limonoids and triterpenes, but also several other compounds, such as monoterpenes, sesquiterpenes, steroids, and other metabolites. Their structures are shown below, and their names and corresponding plant and biological activity sources are compiled in the tables.

### 2.1. Aliphatic Acid and Alcohol

Fatty acids are hydrocarbon chains with a carboxyl group at the end of the molecule and play a number of key roles in metabolism [18]. The main sources of fatty acids are vegetable oils, dairy products, meat products, grain, and fish oils [19], but they were identified in the bark and sapwood oil of *C. odorata* L. Oleic (**2**) and linoleic acids (**3**) are most common in plants, while stearic acid is more common in animals, and a minor component in plants [19]. Stearic (**1**), oleic, and linoleic fatty acids and n-octacosanol alcohol (**4**) were identified in the genus *Cedrela* (Table 1). Fatty alcohol is a class of aliphatic hydrocarbons containing one or more hydroxyl groups and are most prominent in terrestrial plants with C26 to C30 chain lengths [20].

### 2.2. Flavonoids

Flavonoids are a secondary metabolite class, also known as polyphenol antioxidants, found naturally in plants. Moreover, they are widely disbursed throughout plants and gives the flowers and fruits of many plants their vibrant colors [22]. However, De Paula et al. [23] and Da Silva et al. [24] reported the absence of proanthocyanidins—also known as tannins, whose building blocks can be catechin or epicatechin [25,26]—from *C. odorata* L. and their presence in *Toona ciliata*. Thus, “these constituents could have been *trans*located from the Toona stock to the *Cedrela* graft” [24] (p. 1085).

Considering that a review of the latest studies of the composition of *Cedrela* species found isolated flavonoids, further studies on the composition of *Cedrela* are important. Flavonoids such as catechin (**5**) and luteolin (**6**) identified in the genus *Cedrela* are shown in Table 2.

### 2.3. Tocopherol

Tocopherols constitute members of the vitamin E group, essential dietary components in mammals, and are associated with a decreased risk of many diseases [29]. Their biosynthesis is potentially derivable from the oxidation of phenolic compounds to quinones, and isoprenoid side-chain added (four isoprene units) [18]. The terpenoid quinone (**7**) identified in the genus *Cedrela* is shown in Table 3.

### 2.4. Terpenes

#### 2.4.1. Monoterpenes

Monoterpenes are a secondary metabolite class usually identified in the oils that can be obtained from different parts of many plants. Biosynthetically, these compounds can be steam from the condensation of two isoprene units [31]. The monoterpenes (**8**–**32**) identified in the genus *Cedrela* are shown in Table 4.

#### 2.4.2. Sesquiterpenes

Sesquiterpenes are a secondary metabolite class, whose chemical structures are formed from three isoprene units [18]. This class is mainly found in essential plant oil. The few studies about the essential oil composition of *Cedrela* species have shown that sesquiterpenes were found in abundance [21,32,33,34].

Furthermore, according to Ogunwande et al. [33], there is a qualitative or quantitative expressive difference in essential oil composition from different botanic parts of *Cedrela* species. The authors affirmed that sesquiterpene hydrocarbons are present in the essential oil of leaves, while oxygenated derivates are predominant in steam bark essential oil. The sesquiterpenes (**33**–**118**) identified in the genus *Cedrela* are shown in Table 5.

#### 2.4.3. Triterpenoids

##### Triterpenes

Triterpenes, one of the largest classes of natural plant products, are initially formed from squalene, following complex enzymatic reactions [40]. However, in addition to the need for specific enzymes for cyclization, it also depends on the conformation of squalene, yielding mainly oleanane, ursane, lupane, and dammarane carbon skeletons [41]. Triterpenes have a range of biological effects and are used in the prevention and treatment of some diseases [42]. The triterpenes (**119**–**151**) identified in the genus *Cedrela* are shown in Table 6. 

##### Cycloartanes

Cycloartanes are triterpenoids and key intermediates in the biosynthesis of phytosteroids, which contain a cyclopropane ring generated by the inclusion of carbon from the methyl at C-10 catalyzed by cycloartenol synthase enzymes [18,53]. The Cycloartanes (**152**–**154**) identified in the genus *Cedrela* are shown in Table 7.

##### Steroids

Phytosterols are biosynthetically derived from cycloartenol and are characterized by the lack of methyl groups at C-4 and C-14 [54]. Plants have complex and diversified sterol compositions, which are structural components of biological membranes, and that should be a key element of many cellular processes [55]. The steroids (**155**–**162**) identified in the genus *Cedrela* are shown in Table 8.

Limonoids are a secondary metabolite class of highly oxygenated modified triterpenes. This compound is biosynthesized by the acetate–mevalonate pathway and is also known as a tetranorterpenoid because its biosynthesis occurs through oxidative processes of side-chain degradation, resulting in the loss of four carbon atoms and the formation of a furan ring [15,56,57].

The chemical structure formed after the previously mentioned oxidation processes is 4,4,8-trimethyl-17-furanyl steroid, which is considered a precursor of limonoids [14,58]. All compounds of the limonoid class containing this basic structure are derived from different oxidation degrees and rearrangements that give rise to different skeletons [58]. The limonoids (**163**–**215**) identified in the genus *Cedrela* are shown in Table 9.

Limonoids are divided into ten classes, including fifteen types based on different seco- styles, carbon-chain styles, and ring types [16]. This classification was presented by Fang et al. [16], who sought to identify the class and type of limonoids isolated from the *Cedrela* species. Table 10 shows the analysis performed with 55 chemical structures of limonoids isolated from different *Cedrela* species found in this review, showing the different seco-styles and carbon frameworks of these limonoid types. 

As depicted in Table 10, mexicanolide (41.5%), gedunine (17.0%), and obacunol (13.2%) were the main limonoid types found. Thus, it was possible to identify that *Cedrela* limonoids are biosynthesized along only one route, giving rise to the ring-D lactone derivatives and then to the mexicanolide type, characteristic of Swietenioideae [10,23]. 

Da Silva et al. [10] report part of a biogenetic map for limonoids featuring all structural types found in Meliaceae. Route 1.1.2 shows the B,D-seco class, which is characteristic of Swietenioideae, as previously mentioned. This route is related to the most commonly found limonoid class in the genus *Cedrela*. As shown in Table 11, other classes were found; one can affirm that they are associated with the B,D-ring class as biosynthetic intermediaries. 

Since there is no mexicanolide type limonoid in the *Toona* genus, this may not be inserted in the Swietenioideae subfamily with *Cedrela* [10,12,13]. It must be emphasized that the chemical structure of *Toona* limonoids isolated until now present with all intact rings or ring-D intact and A,B-seco rings, which are characteristic of Melioideae subfamily and justify the insertion of *Toona* genus in this subfamily [10,23].

## 3. Biological Activities

As previously shown, 215 compounds were identified or isolated from the genus *Cedrela*. Biological activity surveys were found and reported a few applications for compounds or extracts of this genus including potential oxidants (*C. odorata* [28]), Hsp90a inhibitors (*C. odorata* [45]), PTP1B inhibitors (*C. odorata* [52]), anti-cancer compounds (*C. odorata* [45,49]), trypanocidal compounds (*C. fissilis* [27]), antimalarial compounds (*C. odorata* [70,71]), antifeedant compounds (*C. odorata* [65,72]), insecticidal compounds (*C. fissilis* [42,67]; *C. salvadorensis* [62]), and plant growth inhibitors (*C. salvadorensis* [60]; *C. dugesii* [66]). In this part of the review, we will present the reports from studies on extracts from the genus *Cedrela* and their active compounds.

### 3.1. Antioxidant Activity

Solvent extracts of *C. odorata* stems were tested for free radical scavenging activity in a model reaction with stable 2,2-diphenyl-1-picrylhydrazyl radical (DPPH) by Rashed [28], according to a standard protocol by Saha et al. [85]. The dichloromethane extract presented better activity than other solvent extracts tested (85.45% DPPH free radical scavenging effect).

Although some compounds (**6**, **120**, and **121**) were isolated from the most active extract, the oxidant potential was not tested for these compounds. Nevertheless, the authors carried out an investigation on the presence of phytochemicals in the solvent extracts of *C. odorata* stems as a reasoned justification for active extracts. Therefore, flavonoids, steroids, and triterpenes were detected, compounds with good antioxidant effects that could be due to the hydroxyl groups present in the compounds found and which were present in ample amounts in the extract.

### 3.2. Hsp90a Inhibitors and Anti-Cancer Activity

Compounds (**155**, **156**, **125**, **126**, **168**, **173**, **176**, **198**, **204**, **206**, **211**, **212**, **213**, and **215**), isolated from *C. odorata* leaves, were assayed to evaluate the ability to inhibit Heat Shock Protein 90 (Hsp90) activity, adopting a multidisciplinary approach with Surface Plasmon Resonance (SPR) measurements and biochemical and cellular assays. Hsp90, considered a molecular chaperone, plays an essential role in many cellular processes, including the stabilization of a range of proteins necessary to the survival of many kinds of cancer [86]. As a result, the identification of an inhibitor of Hsp90 is the main target of the “development of more effective drugs for the treatment of various types of multidrug-resistant malignancies” [45].

In order to verify compounds with affinity for Hsp90, SPR was carried out which showed that some compounds (**125**, **168**, **176**, **211**–**213**, and **215**) interacted with the protein. Nonetheless, three of them showed better affinity towards the chaperone (**211** (*K*_D_ = 14 ± 5 nM; *k*_a_ = 429 ± 8 (nM × s)^−1^; *k*_d_ = 6 ± 1 (1/s) × 10^−3^), **213** (*K*_D_ = 18 ± 6 nM; *k*_a_ = 16 ± 3 (nM × s)^−1^; *k*_d_ = 0.3 ± 0.1 (1/s) × 10^−3^), and **215** (*K*_D_ = 15 ± 2 nM; *k*_a_ = 467 ± 26 (nM × s)^−1^; *k*_d_ = 7 ± 1 (1/s) × 10^−3^)).

Comparing the thermodynamic (*K*_D_) and kinetic constant (dissociation—*k*_d_ and association—*k*_a_) values for these three compounds, the authors suggest that they could interact with the protein from a different approach. Thus was suggested because compound **213** presented a low *k*_d_ value, whereas compounds **211** and **215** presented better *k*_a_ values and was confirmed when the limited proteolysis-mass spectrometry-based approach was performed, making it possible to lift and confirm the hypothesis that compounds **211** and **215** prevented the proteolytic digestion of Hsp90a at Lys564 and Lys614, which are two residues located in the protein C-terminal; C-terminal being the point of interaction of these compounds with Hsp90.

Possible biological effects for compounds **211** and **215** were evaluated and provided an understanding that the activity of the compounds was related to the Tetrahydrofuran ring at C-17, which is responsible for their more efficient pattern of hydrogen bonds and for some specific hydrophobic contacts with Glu477chainA.

Based on the possibility of compounds **211**, **213**, and **215** interacting with Hsp90, inhibiting its activity, the cytotoxic activity of these compounds was assessed against epithelial carcinoma cell lines (human HeLa) and breast carcinoma cell lines (MCF-7) by Microculture of Tetrazolium (MTT) proliferation assay. Results showed that for the two cell lines tested, compound **211** inhibited cell proliferation (IC_50_ = 10.1 ± 0.2 µM in HeLa and 51.3 ± 0.3 µM in MCF-7 cells), in a concentration-dependent manner, whereas compounds **213** and **215** showed no significant results (IC_50_ > 100 µM for two compounds in both cell lines).

Still considering the anti-cancer activity, in 2018, Wan et al. [49] investigated, through a number of bioassays, the action mechanism of the anti-cancer effect of compound **127**, being known that this compound demonstrates oxidant potential, being able to interrupt the cell cycle of cancer cell lines MCF-7, NCI-H460, and A375-C5 [87]. Nonetheless, it is not known if compound **127**, as well as other limonoids, could cause autophagy and other cellular responses in carcinoma cells.

Compound **127**, isolated from *C. odorata*, inhibited cell growth in a time-dependent and concentration-dependent manner by induced cell death, which could have been due to apoptosis or autophagy. Flow cytometry results presented little or no apoptosis in the tested concentrations (8 µM, 16 µM, and 32 µM), showing that this is not the reason for the inhibition of cell proliferation.

Furthermore, it was noted that compound **127** induced the accumulation of excess reactive oxygen species (ROS), which can give rise to other molecular responses such as autophagy. This was observed as compound **127** increased autophagy marker levels early and provoked the conversion of Light Chain 3 (LC3) -I to LC3-II, which directly indicates autophagy. Adding these results to those observed in flow cytometry, the authors suggested that compound **127** induced autophagy.

With the purpose of evaluating the anti-cancer capacity of compound **127**, Wan et al. [49] verified through western blot assay based on Lu et al. [88] the regulatory roles of compound **127** in ROS/mitogen-activated protein kinase kinase (MEK)/extracellular signal-regulated kinase (ERK) signaling pathways and effects of these on the autophagy, migration, and invasion. N-acetyl-l-cysteine (NAC) was used as a ROS remover was able to suppress ROS production, and the role of compound **127** in MEK/ERK signaling. On the other hand, U0126 used as a MEK/ERK inhibitor did not inhibit ROS production, although a decrease in MEK/ERK activity was observed. Thus, it was possible to identify that compound **127** activated the MEK/ERK signaling, playing a crucial role.

The effect of compound **127** in vivo was also tested against MKN45 cell xenograft tumors and patient-derived xenograft (PDX) tumor models. This compound was able to inhibit tumor growth, where BALB/C nude mice did not present toxic syndromes, diminished activity, or death, and there were no dramatic changes in body weight with either tumor model. Moreover, and very importantly, the Ki67 expression was inhibited, which indicated that the tumor proliferation was inhibited.

### 3.3. Protein Tyrosine Phosphatase 1B Inhibition

The potential of compounds **136** and **137** isolated from *C. odorata* twigs and leaves [52] for the inhibition of protein tyrosine phosphatase 1B (PTP1B) was evaluated through a bioassay according to Zhang et al. [89]. These compounds (**136** IC_50_ = 13.09 µg/mL; **137** IC_50_ = 3.93 µg/mL) presented significant PTP1B inhibition potential. PTP1B is an important therapeutic target of some metabolic disorders such as diabetes and obesity, and assays performed by Elchebly et al. [90] with PTP1B-deficient mice showed enhanced insulin sensitivity and resistance to diet-induced obesity.

### 3.4. Trypanocidal Activity

Crude extracts of different botanical parts, limonoids (**180**–**182** and **207**), and triterpenes (**119**, **121**, **122**, **127**–**129**, and **131**–**133**) from *C. fissilis* were tested for trypanocidal activity by Leite et al. [27] with a procedure for in vitro assays on blood trypomastigote forms of *Trypanosoma cruzi*, as previously described by Ambrozin et al. [91].

Results of the bioassays with the extracts showed that dichloromethane root extracts were the most active, reducing the number of parasites in the infected mouse blood by 97.4%; 11 other extracts were significantly active at the higher concentration tested (lysis% ≥ 50), while nine others presented lysis in the 60% to 80% range. Assays were performed with extract concentrations of 4 mg/mL.

Knowing the active extracts, the authors carried out compound isolation of dichloromethane extracts from leaves (70.8%), fruits (75.4%), stems (70.3%), and roots; ethanol extracts from fruits (69.3%) and stems (58.0%), and also one inactive extract, root hexane (33.8%). From the isolated compounds, 14 were tested at 50, 100, and 250 μg/mL concentrations.

Triterpenes were the most active compounds. Tirucallane triterpenes (**127**–**129**), isolated from methanol stem extract, stood out for their high activity that could be justified by the presence of the hydroxyl group at C-3 and the cyclization of the side chain to a dihydroxy-tetrahydrofuran. Leite et al. [27] reported that compound **127** showed better activity than the positive control, demonstrating its potential to act as a substitute for gentian violet, which presents collateral effects; however, a toxicity assessment must be carried out. Moreover, *C. fissilis* proved to be efficient in the production of compounds that can be used to control Chagas disease.

### 3.5. Antimalarial Activity

Ethanolic extracts of 22 Meliaceae species, including *C. odorata*, *C. salvadorensis*, and *C. fissilis,* were tested for antimalarial activity by MacKinnon et al. [70] against two clones of *Plasmodium falciparum*: D6 (chloroquine-sensitive) and W2 (chloroquine-resistant); it was used by Desjardins et al. [92] in an antimalarial screening bioassay.

Extracts of all parts tested of *C. fissilis* were inactive for both clones (IC_50_ > 20 µg/mL), whereas *C. odorata* wood and *C. salvadorensis* leaf extracts were two of the most active against clones D6 and W2 (IC_50_ < 20 µg/mL). However, compared to the other extracts tested, *C. odorata* showed higher activity. IC_50_ values for these *Cedrela* species are presented in Table 11 [69].

An EtOH extract of *C. odorata* wood from Belize was more active than extracts of the same species from Costa Rica and Togo; Effective Concentrations (EC) of these extracts for W2 clone were, respectively, 1.10, 1.25, and 3.26 µg/mL.

Toluene extraction was also carried out, resulting in a cleaner extract and with a slightly higher content of compound **181** than EtOH extracts. In addition, these extracts were found to be active, with better efficacy against the W2 clone. EC values were: Togo, 2.77 µg/mL; Costa Rica, 0.70 µg/mL; and Belize, 0.65 µg/mL.

Previous phytochemical studies showed that limonoid contents in Meliaceae species present antimalarial activity, making it possible to suggest that the limonoid constituents in these extracts could be responsible for the activity. Thus, there was the recognition that compound **181** was present in *C. odorata* extract and was especially active, requiring further investigation.

**Figure 2:**
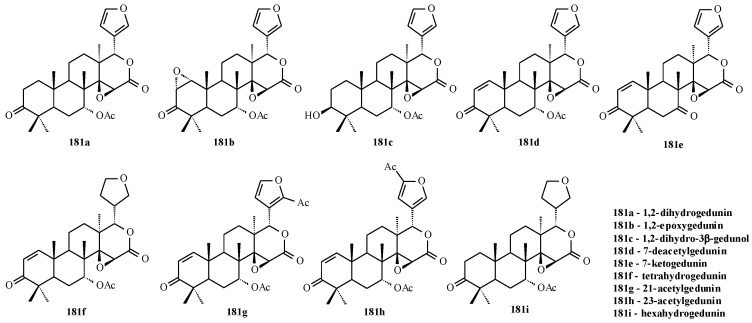
Gedunin (**181**) semi-synthetic derivatives [70].

Accordingly, this study also carried out assays to examine the antimalarial activity of compound **181** and nine synthetic derivatives (**181a**, **181b**, **181c**, **181d**, **181e**, **181f**, **181h**, and **181i**) to define structure-activity relationships for this molecule. Figure 2 illustrates the chemical structures of derivatives from compound **181**.

Regarding compounds with antimalarial activity, none of the compounds showed better activity than compound **181**. However, the study added knowledge to topics related to the structure-activity relationships for antimalarial activity of D-seco limonoids.

Based on assays with these structurally modified compounds, it proved possible to identify three systems in the chemical structure, which could be associated with the activity of compound **181**: α,β-unsaturated ketone moiety in ring A, 7-acetate of ring B, and the furan ring.

Comparing compound **181** (IC_50_: D6 = 39 ng/mL − W2 = 20 ng/mL) with compound **181a** (IC_50_: D6 = >10000 ng/mL − W2 = 840 ng/mL), compound **181b** (IC_50_: D6 = 2580 ng/mL − W2 = 980 ng/mL), and compound **181c** (IC_50_: D6 = 4210 ng/mL − W2 = 2440 ng/mL) a decrease in activity was noted when unsaturation was reduced (**181a**), epoxidation to the 1,2-β-epoxy derivative (**181b**), or unsaturation and ketone reductions to 1,2 dihydro-β-gedunol (**181c**). In all these cases, activity against both clones was reduced, mainly D6.

Compounds **181d** (IC_50_: D6 = 2610 ng/mL − W2 = 1280 ng/mL) and **181e** (IC_50_: D6 = >10,000 ng/mL − W2 = >10,000 ng/mL) also showed some activity decrease in both cones. The authors affirmed that this was related to the group present at C-7 because when this group was a ketone group (compound **181e**) it was observed to be inactive.

With respect to the furan ring, it was observed that system less contributed than the unsaturation or the group at C7 for activity. Compounds **181f** (IC_50_: D6 = 2500 ng/mL − W2 = 900 ng/mL), **181g** (IC_50_: D6 = 133 ng/mL − W2 = 39 ng/mL), **181h** (IC_50_: D6 = 832 ng/mL − W2 = 156 ng/mL), and **181i** (IC_50_: D6 = 10,000 ng/mL − W2 = 2130 ng/mL), when compared to the rest, showed that activity was reduced to a lesser magnitude than other derivate compounds.

Finally, the majority of the compounds showed activity against chloroquine-resistant clones, with compound **181** having a lower IC_50_ value of than chloroquine. Furthermore, the cytotoxicity of these compounds was evaluated using KB cells, as described previously by Likhitwitayawuid et al. [93], and an in vivo assay of compound **181**. For the cytotoxicity assay, compounds **181** (2300 ng/mL), **181g** (9400 ng/mL), and **181h** (10,900 ng/mL) presented some cytotoxicity; however, less than drugs usually used to treat malaria, chloroquine (17,400 ng/mL), quinine (>20,000 ng/mL), mefloquine (3500 ng/mL), and artemisinin (>20,000 ng/mL), suggesting a possible new antimalarial. For the in vivo assay, where compound **181** was administered orally or subcutaneously to mice infected with *Plasmodium berghei*, during a four-day test, there was no inhibition of parasitemia. Nonetheless, according to the authors, the biological potential of compounds requires further study because of the focus on traditional remedies in Africa.

**Figure 3:**
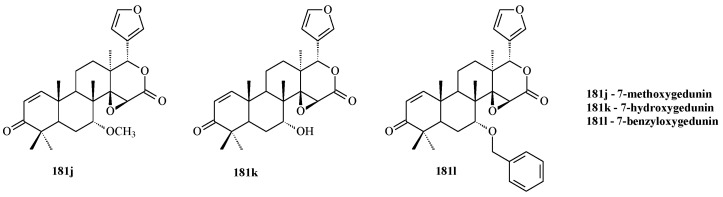
Gedunin (**181**) semi-synthetic derivatives [70].

Considering the low in vivo activity present in the MacKinnon et al. [70] study, Omar et al. [71] mentioned possible reasons, for instance: “poor solubility and uptake of the drug due to its lipophilicity”; “first-pass metabolism of gedunin by cytochrome P450 enzymes of the small intestine, which reduce plasma levels of drugs; and hydrolysis of gedunin to the inactive and unstable metabolite, 7-deacetylgedunin” [71] (p. 135). Thus, Omar et al. [71] carried out compound co-precipitation with polyvinylpyrrolidone (PVP) for oral administration; compounds were tested alone and in binary treatment, where the compound was administered with dillapiol, a cytochrome P450 inhibitor. Another approach was that the semi-synthetic derivatives (compounds **181j**, **181k**, and **181l**, Figure 3) had changes in the labile acetoxy group at the C-7 moiety because it was prone to hydrolysis and subsequent rearrangement.

Compound **181** (50 mg·kg^−1^·day^−1^) was orally administered to CD-1 mice with parasite clearance of 44.6 ± 5.6%. Although this is the first in vivo efficacy reported, a follow-up dose-response study (25–100 mg·kg^−1^·day^−1^) showed poor parasite clearance at other concentrations, and no clear dose-response was observed.

The mean blood concentration of compound **181**, after 3 and 6 h, was found to be 6.2 µg/mL. A preliminary pharmacokinetic study was performed with Sprague-Dawley rats to identify the concentrations of compound **181**. Poor absorption and rapid clearance from the blood were clearly observed; thus, a binary treatment was conducted, combining dillapiol with semi-synthetic derivatives.

Oral administration of compound **181** and dillapiol in CD-1 mice infected with *P. berghei* showed better parasite clearance (79.0% ± 5.3%), in which the increase was dose-dependent with compound **181** doses up to 50 mg·kg^−1^·day^−1^, with dillapiol held constant at 25 mg·kg^−1^·day^−1^. In addition, the mean blood concentration of compound **181** increased to 13.1 µg·mL^−1^, which could be related to cytochrome P450 inhibition in the small intestine, although the dillapiol penetrant properties may play a role. Regarding the toxicity of the binary treatment, preliminary studies show that there were no significant differences in total body or organ weight, gross anatomy, or three liver enzyme levels relative to the control group; nevertheless, chronic toxicity studies need to be carried out.

Among the semi-synthetic derivatives, only compounds **181j** and **181l** showed antiplasmodial activity, although it was lower than the activity of compound **181**. The in vivo bioassay was accomplished with compound **181j**, due to lower molecular weight, and was relatively active. This compound showed better activity than compound **181** when given alone and in binary treatment, with a clearance of 67.5% ± 1.3% (50 mg·kg^−1^·day^−1^) and 80.7% ± 5.1% (50 mg·kg^−1^·day^−1^ of compound **181j** and 25 mg·kg^−1^·day^−1^ of dillapiol), respectively. When compared to a conventional drug used in antimalarial therapy (quinine = 60 ± 10%—10 mg·kg^−1^·day^−1^), these compounds showed promising results, which could contribute to the development of effective phytomedicines based on traditionally used antimalarial plants.

### 3.6. Antifeedant Activity

Limonoids **170**–**175**, **199**, and **203**–**207** were isolated from *C. odorata* stem bark, and their antifeedant activity briefly tested against the third-instar larvae of *Spodoptera littoralis* (Boisd.) by Kipassa et al. [65] with Wada and Munakata’s [94] test methodology. Only limonoid **203**, mexicanolide type, was active at the 500 ppm concentration. The authors point out that at a 1000 ppm concentration, the obacunol type (**170**–**175**) showed weak activity while other mexicanolide (**199** and **203–207**) types showed moderate activities.

This activity was also tested by Omar et al. [72] for compound **181**, isolated from *C. odorata* wood, against the rice weevil, *Sitophilus oryzae* (L.) using a method developed by Xie et al. [95]. Four concentrations (0%, 0.05%, 0.25%, and 0.50% *w*/*w*) were tested, however the authors reported that the compound was active at 0.50% (*w*/*w*), with a consumption of diet value of 31.2% ± 6.3% control ± SEM.

### 3.7. Insecticidal Activity

Triterpenes (**121** and **122**) and a limonoid (**207**) were isolated from *C. fissilis* roots. Insecticidal activity was tested against *Atta sexdens rubropilosa* by Leite et al. [42], and this survey was a continuation of the tests of Bueno et al. [96] where *C. fissilis* root extracts showed activity on leaf-cutting workers, *A. sexdens rubropilosa*.

Compounds (100 μg/mL) tested were administered in an artificial diet (0.4–0.5 g per dish), according to Bueno et al. [97] and offered daily. A control diet was used with the addition of compounds or without. The ants were followed for 25 days, and the number of dead ants was registered daily.

Among the compounds tested, triterpenes and limonoid of *C. fissilis* showed significant activity when compared to the other compounds and control. The authors reported that activity could be related to triterpenes and these compounds tested are promising in controlling leaf-cutting ants.

Limonoids (**178**, **180**, **181**, **182**, **184**, and **185**) from *C. fissilis* roots and leaves showed insecticidal activity against *A. sexdensrubropilosa* ants and was also tested by Ambrozin et al. [67] using the same previously mentioned methodology. The limonoids tested and the control showed the same survival medium time; however, compound **184** presented a significant difference to the control according to the log-rank test (*p* < 0.05). All these limonoids are of the gedunin type, and the authors affirmed that this type does not seem to be highly active against these ants. The authors further suggest that the toxicity of *C. fissilis* extract could be associated with the synergism between limonoids and other compounds in the extracts.

The insecticidal activity was also tested by Jimenez et al. [59]; compound **164** present in *C. salvadorensis* had an effect against *Ostrinianubilalis* (the European corn borer). This compound was administered in an artificial diet with a concentration of 5 or 50 ppm for neonate larvae, after which thirty of them were *trans*ferred to separate vials with corresponding diet cubes. On the 20th day, the following were observed: weight gains, mortality, time to pupation and adulthood, the weight of pupae and adults, the mortality of larvae, and adult deformities.

Compound **164** inhibited growth at the larval stage; on day 20, this compound clearly showed a reduction in larval growth, moderate larval mortalities (<37%), and delays in time to pupation were observed in 5 ppm cedrelanolide-treated males, 50 ppm treated females when compared to the humilinolides from *Swietenia humilis*. Regarding survival to the adult stage, as compared to the pupal stage, compound **164** presented no further reductions. The authors affirmed that, similar to other limonoids, the reduction in insect growth from compound **164** and other limonoids evaluated can be associated with a combination of antifeedant action and post-digestive toxicity.

The insect growth regulatory activity of an epimeric mixture of compounds **164**, **177**, and **178**, and **181** from *C. dugessi* and *C. salvadorensis* heartwood was evaluated against *Spodoptera frugiperda* by Céspedes et al. [62].

**Figure 4:**
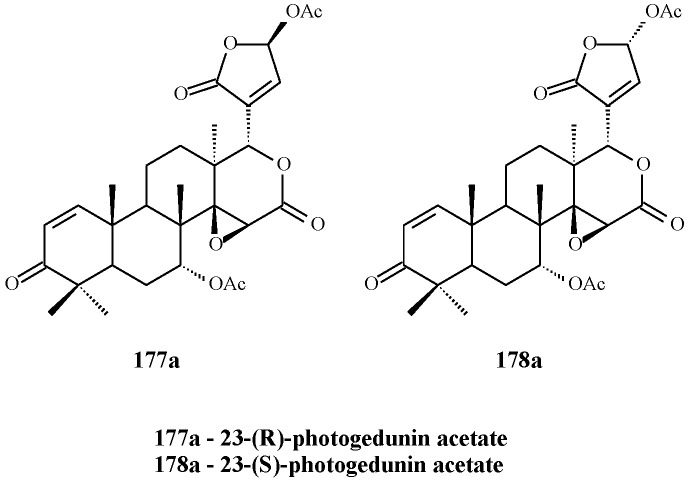
Derivatives of compounds **177** and **178** by Céspedes et al. [75].

Previously mentioned compounds and an anomeric acetate mixture (compounds **177a** and **178a** [75], Figure 4 shows these derivatives’ chemical structure) were dissolved in ethanol and administered at different concentrations (ranging from 5 to 50 ppm) in an artificial diet prepared according to Mihm [98]. Only one neonate first instar larvae was placed in contact with the diet, for seven days, with different concentrations and compounds, inclusively with a positive control (toosendanin). After seven days, surviving larvae were transferred to separate vials containing a fresh stock diet for 21 days, and weight gains, mortality, and other lifecycle measurements were evaluated.

At the larval stage, all tested compounds at 52 ppm, except cedrelanolide, inhibited growth. At lower concentrations, only compounds **177a** and **178a** showed higher insecticidal activity, with 83.3% larval mortality at 10 ppm. After 21 days, it was observed that compound **181** showed better results than the positive control, toosendanin, at 5 ppm.

All compounds reduced the percentage of larvae that reached the pupation stage, compared to the control. On the other hand, none of them significantly reduced pupation time. Nonetheless, compounds **177a** and **178a** showed a drastic reduction in adult emergence percentages at all concentrations tested.

The action mechanism of limonoids is not clear; however, it could be related to physiological effects, with the oxygenated function at C-23 being necessary for the activity of compounds **177**, **178**, **177a**, **178a**, and **181**; the authors suggest, based on Champagne et al. [99] and Isman et al. [100], that there could be a combination between antifeedant action and/or post-digestive toxicity, as found for other limonoids. Moreover, although the compounds tested showed activity compared to the positive control, except **164**, suggesting additional production, none of them demonstrated the outstanding activity of azadirachtin [59].

### 3.8. Plant Growth Inhibitory Activity

Céspedes et al. [60,66] carried out assays to investigate the phytotoxic properties of compounds from different *Cedrela* species, on germination, root, and shoot development, and the respiration of seeds of two monocots (*Lolium multiflorum* and *Triticum vulgare*) and two dicots (*Physalis ixocarpa* and *Trifolium alexandrinum*). With similar methodologies, these studies differed mainly in the compound tested, bioactivity-guided isolation [66], and verification of possible effects on the photosynthesis system presented by Céspedes et al. [60].

Céspedes et al. [66] accomplished bioactivity-guided isolation, in which heartwood parts of *C. dugesii* were extracted with CH_2_Cl_2_ against two monocots seeds. The authors were able to verify the most active fraction (F3—*L. multiflorum* = IC_50_ 24.0 μg/mL; *T. vulgare* = IC_50_ 23.0 μg/mL) and to arrive at the compound responsible for the activity, which was an epimeric mixture of compounds **177** and **178**.

The epimeric mixture of compounds **177** and **178** and semi-synthetic acetylated derivatives were reported in Céspedes et al. [75] (compounds **177a** and **178a**, Figure 4), and were assessed for phytotoxic effects on monocot and dicot seed germination. Both compounds and mixtures showed similar inhibition profiles; however, monocot seeds (*T. vulgare* and *L. mutlifloreum*) were most sensitive at 50 μM and 500 μM, respectively, for photogedunin epimeric acetates, presenting with an almost 100% inhibition. Comparing concentrations that inhibit 50% of seed germination (GI_50_), the low values for photogedunin acetate mixtures showed that they were better inhibitors. Moreover, this comparison allowed speculation that the low activity of the mixture of compounds **177** and **178** could be related to the hydrophilicity of the free C-23, where the hydroxyl group makes it difficult for photogedunin to reach the target.

As for growth inhibition, a dose-dependent pattern was shown, which stimulated or inhibited germination for both seeds. In the case of monocot seeds, there were inhibitory effects of the photogedunin mixture on root and coleoptile development, whereas, in dicot seeds, the promotion of root and hypocotyl development was observed. Nonetheless, according to the authors, the root developed to a greater extent than coleoptile or hypocotyl development (the lowest median infective dose (ID_50_) values). Higher ID_50_ values showed that dicot seed growth was more sensitive to photogedunin acetate mixture.

With respect to dry weight and respiration, these decrease with increases in photogedunin mixture concentration; however, the *L. multiflorum* respiration rate was one exception, as it was more sensitive to inhibition while *T. alexandrinum* showed higher resistance. This suggests that the photogedunin mixture acted as an uncoupler to phosphorylation at low concentrations, but at higher concentrations it either inhibited energy transduction or the respiration redox enzymes. It was shown that the photogedunin epimeric mixture was more selective and potent towards monocots than dicots.

The authors presented graphics comparing the inhibition of the mixtures tested, showing that the acetylated derivative was ten times larger than the photogedunin mixture, which could be active in vivo, and also suggests that compounds and mixtures can have more than one target of interference because germination inhibition occurs at lower concentrations than respiration.

On the other hand, as a complement to the investigations of Lotina-Hennsen et al. [101] on biologically active limonoids with potential herbicidal properties, Céspedes et al. [60] carried out assays to evaluate phytotoxic properties of compound **164** from the heartwood of *C. salvadorensis* against the same species of monocot and dicot seeds presented in Céspedes et al. [66].

In general, if compared to the Céspedes et al. [66] assays with **177** and **178**, **164** showed a better inhibition profile. Concentration-dependent, **164** inhibited respiration, germination, shoot and root elongation, and dry weight of the tested species, especially monocot species. It is worth pointing out that *L. multiflorum* was shown to be the most susceptible to this compound, with GI_50_ 120.6 μM, and causing 100% germination inhibition at 300 μM.

In the case of growth inhibition, no significant influence on *T. vulgare* growth (100% inhibited at 500 μM) was observed and a lower inhibition effect on *L. multiflorum* (300 μM), indicating that the mechanism of action of compound **164** could be different for germination and respiration. The authors affirmed that, based on a pre-emergency assay, oxygenated functions of the molecule played an inhibition role in germination due to lipophilicity or the hydrophilicity of the oxygenated function of compound **164** (at physiological pH), making it difficult for compound **164** to reach the target site.

Regarding interference in photosynthesis, the authors evaluated the effects of compounds on different photosynthetic reactions. Based on the obtained results and compared to other group studies, Céspedes et al. [60] assert that compound **164** inhibits the proton chain (IC_50_ = 34.9 µM) and further suggests that it can inhibit all the electron transport chain of water to methyl viologen at the phosphorylation of photosynthesis of water to methyl viologen (inhibited totally at 60 µM; IC_50_ = 29.5 µM). The authors associated electron transport inhibition to the oxygenated functions of compound **164**, which significantly increased the inhibition potency that was similar to that of the Hill’s reaction inhibiting effect of some commercial herbicides.

## 4. Conclusions

This article presents 215 chemical compounds, including chemical structures, found in quali or quantitative phytochemical studies on *Cedrela* species from early 1960 to 2020. It was possible to observe that of 18 species, the most commonly studied species was *C. odorata* L.; however, other species such as *C. fissilis* Vell., *C. salvadorensis* Standl., *C. dugesii* S. Watson, *C. montana* Moritz ex Turcz., *C. angustifolia* DC. and *C. oaxacensis* C.DC. and Rose were also studied. It can also be noted that there are few studies about it; thus, these species and species that still remain unresearched should be considered in further phytochemical and pharmacological studies, which may be a promising direction in the search for new compounds with pharmacological properties.

**Scheme 1:**
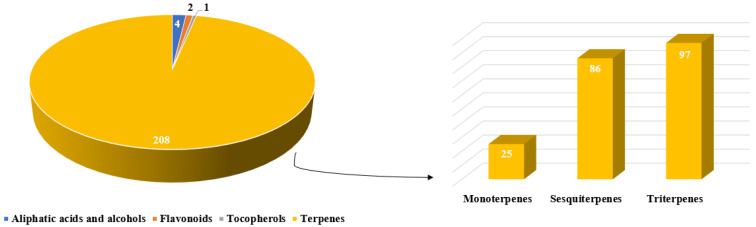
Compound classes isolated or indentified from *Cedrela* species.

To date, as shown below in Scheme 1, four aliphatic hydrocarbons, three phenolic compounds, and 208 terpenes have been identified or isolated. For the Terpene class, which is the biggest class, 25 monoterpenes, 86 sesquiterpenes, and 97 triterpenoids have been found.

**Scheme 2:**
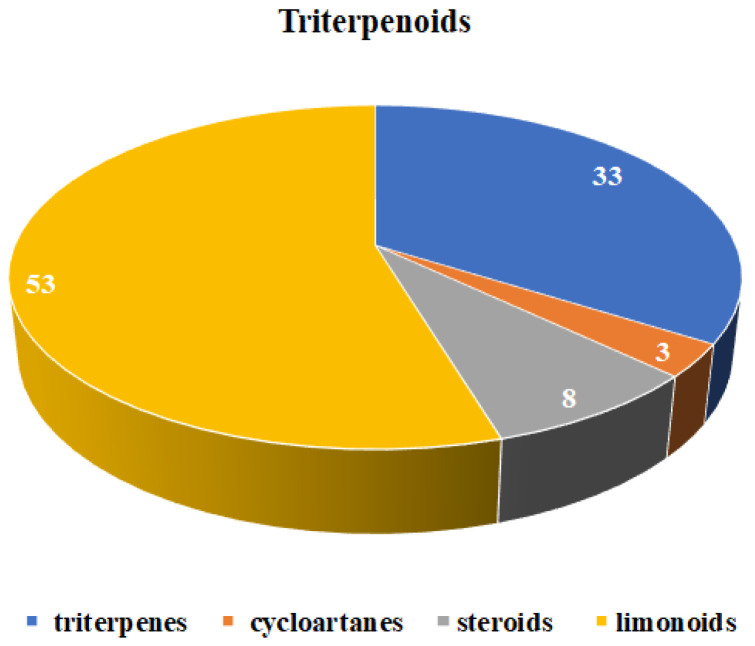
Kinds of triterpenoids isolated from *Cedrela* species.

With respect to triterpenoids identified in the genus *Cedrela*, these belong to triterpenes, cycloartanes, steroids, and limonoids, and 33, three, eight, and 53 of these classes were found, respectively, as pictured in Scheme 2, below.

**Scheme 3:**
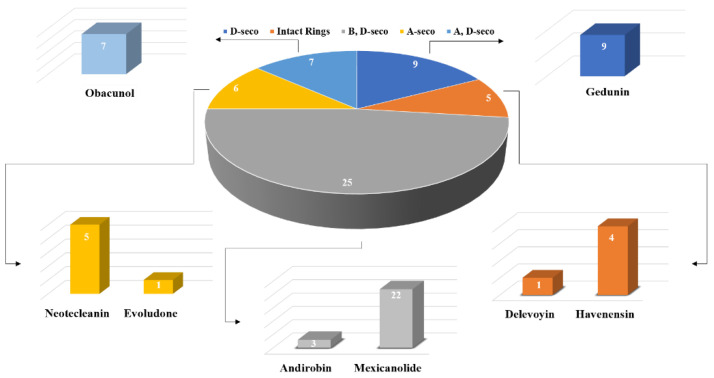
Class and types of limonoids found in the genus *Cedrela.*

As limonoids are a characteristic class of the Meliaceae family, these compounds found in *Cedrela* species were classified based on Fang et al. [16]. Scheme 3 shows the classes and types of limonoids found in the genus *Cedrela*. As depicted, B,D-seco was the main class found, with 25 limonoids, most of them mexicanolide type (22). Other limonoid types were also found but in much smaller quantities, such as gedunine (9), obacul (7), neotecleanin (5), evodulone (1), delevoyin (1), havenensin (4), and andirobin (3). One degraded limonoid was also found; nonetheless, Fang et al. [16] did not relate this limonoid type to any class. These authors suggest that this type could be the origin of degradation of intact limonoids since it possesses a furan ring and the C and D ring portions of the original limonoids.

The presence of mexicanolide, obacunol, and gedunin types indicates that *Cedrela* limonoids are ring-D lactone derivatives and of the mexicanolide type, which is characteristic of the Swietenioideae subfamily [10,23].

Biological activities were also compiled in this article. As expected, due to the known insecticidal activity of limonoids, more articles were found reporting this activity, antifeedant, and growth inhibition of insects. However, of the limonoids tested, none demonstrated better activity than or compared to azadirachtin.

In the case of activities related to human health, promising results were achieved in the antitripanocidal activity of compound **127** and in the antimalarial activity of compound **181**. Although these results are interesting, more studies must be conducted in order to describe the real efficacy, besides continuing the pharmacological studies. These can be interpreted because of the concentration of these compounds in the botanical parts or in the species, therefore phytochemical studies that use validated analytical methods, or in vitro culturing, aimed at obtaining these secondary metabolites in a sustainable way are required.

Studies using analytical methods could also help to understand if *Cedrela* species are sources of substances that have or have not had their activities reported by the articles mentioned here, or that have already been isolated from other species and have been subject to pharmacological and toxicological studies.

## Figures and Tables

**Table 1:** Aliphatic acids and alcohol isolated from the genus *Cedrela* (1960–2020).

Compound	Plant Species	Plant Part	References
stearic acid (**1**)	*Cedrela odorata* L.	bark and sapwood oil	[21]
oleic acid (**2**)			
linoleic acid (**3**)			
n-octacosanol (**4**)			
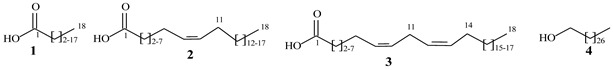			

**Table 2:** Flavonoids isolated from the genus *Cedrela* (1960–2020).

Compound	Plant Species	Plant Part	References
Catechin (**5**)	*C. fissilis* Vell.	Fruits	[27]
	*C. odorata* L. grafted with *Toona australis* (F. Muell.) Harms	Stems	[24]
Luteolin (**6**)	*C. odorata* L.	Stems	[28]
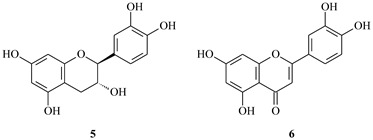			

**Table 3:** Tocopherol isolated from the genus *Cedrela* (1960–2019)

Compound	Plant Species	Plant Part	References
D-α-tocoferol (**7**)	*C. odorata* L.	Leaves	[30]
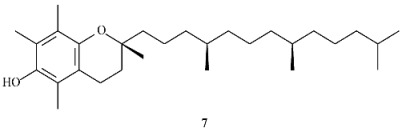			

**Table 4:** Monoterpenes identified from the genus *Cedrela* (1960–2020).

Compound	Plant Species	Plant Part	References
*α*-Pinene (**8**)	*C. odorata* L.	Leaf essential oil	[32]
		Oil from leaves and stem barks	[33]
2-Pentylfuran (**9**)		Leaf essential oil	[32]
Limonene (**10**)		Leaf essential oil	[32]
		Leaf oil	[33]
Bornil acetate (**11**)		Leaf essential oil	[32]
*α*-Thujene (**12**)		Oil from leaves and stem barks	[33]
Sabinene (**13**)			
β-Pinene (**14**)			
Myrcene (**15**)		Stem bark oil	
α-Phellandrene (**16**)			
p-Cymene (**17**)		Oil from leaves and stem barks	
1,8-Cineole (**18**)		Stem bark oil	
Acetophenone (**19**)		Leaf oil	
γ-Terpinene (**20**)		Oil from leaves and stem barks	
*trans*-Sabinenehydrate (**21**)		Stem bark oil	
Terpinolene (**22**)		Oil from leaves and stem barks	
Linalool (**23**)		Stem bark oil	
*trans*-Pinocarveol (**24**)		Stem bark oil	
*trans*-Verbenol (**25**)		Stem bark oil	
Terpinen-4-ol (**26**)		Stem bark oil	
α-Terpineol (**27**)		Leaf oil	
Myrtenol (**28**)		Stem bark oil	
*trans*-Piperitol (**29**)		Stem bark oil	
Cuminaldehyde (**30**)		Stem bark oil	
Thymol (**31**)		Stem bark oil	
α-Terpinylacetate (**32**)		Leaf oil	
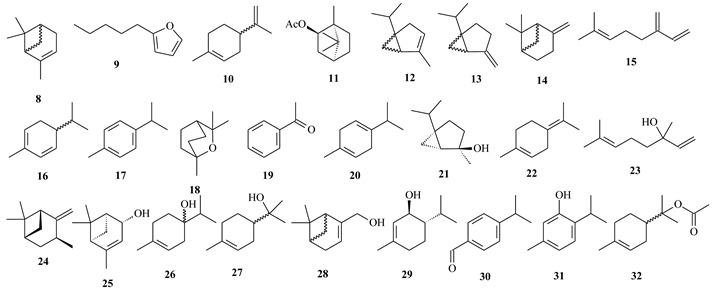			

**Table 5:** Sesquiterpenes identified from the genus *Cedrela* (1960–2020).

Compound	Plant Species	Plant Part	References
Cedrelanol (**33**)	*C. odorata* L.	Leaf essential oil	[35]
	*C. odorata* L.	*-*	[36,37]
Torreyol (**34**)	*C. odorata* L.	Bark and sapwood essential oil	[21]
α-Muurolene (**35**)	*C. odorata* L.	Essential oils from bark, sapwood, and heartwood	[21]
	*C. odorata* L.	Leaves	[38]
	*C. fissilis* Vell.	Stem bark oil	[39]
γ-Muurolene (**36**)	*C. odorata* L.	Bark and sapwood essential oil	[21]
α-Copaene (**37**)	*C. odorata* L.	Essential oils from bark, sapwood, and heartwood	[21]
		Leaf essential oil	[32]
	*C. fissilis* Vell.	Leaf and stem bark oil	[39]
	*C. odorata* L.	Leaves	[38]
α-Cubebene (**38**)	*C. odorata* L.	Heartwood essential oil	[21]
		Leaf essential oil	[32]
	*C. odorata* L.	Leaf oil	[33]
	*C. fissilis* Vell.	Stem bark oil	[39]
		Leaves	[38]
β-Elemene (**39**)	*C. odorata* L.	Essential oils from bark, sapwood, and heartwood	[21]
		Leaf essential oil	[32]
	*C. odorata* L.	Leaf and stem bark oil	[33]
	*C. odorata* L.	Leaves	[38]
		Leaves	[30]
	*C. fissilis* Vell.	Leaf and stem bark oil	[39]
Nerolidol (**40**)	*C. fissilis* Vell.	Leaves oil	[39]
Guayazulene (**41**)	*C. odorata* L.	Essential oils from bark, sapwood, and heartwood	[21]
δ-Elemene (**42**)	*C. odorata* L.	-	[34]
	*C. odorata* L.	Stem bark oil	[33]
(-)-δ-Cadinene (**43**)	*C. odorata* L.	-	[34]
(+)-δ-Cadinene (**44**)			
(*1R,4S*)-Calamenene (**45**)	*C. odorata* L.	Essential oils from bark, sapwood, and heartwood	[21]
		-	[34]
	*C. odorata* L. grafted with *Toona australis* (F. Muell.) Harms	Stems	[23]
		Stems	[24]
β-Caryophyllene (**46**)	*C. fissilis* Vell.	Leaf and stem bark oil	[39]
	*C. odorata* L.	Leaf and stem bark oil	[33]
	*C. odorata* L.	Leaves	[38]
		Leaves	[30]
	*C. odorata* L.	Leaf essential oil	[32]
Aromadendrene (**47**)	*C. fissilis* Vell.	Roots	[39]
	*C. odorata* L.	Leaf oil	[33]
	*C. odorata* L.	Leaves	[38]
		Leaves	[30]
α-Humulene (**48**)	*C. fissilis* Vell.	Leaf and stem bark oil	[39]
	*C. odorata* L. *C. fissilis* Vell.	Leaves	[38]
	*C. odorata* L.	Leaves	[30]
	*C. odorata* L.	Leaf and stem bark oil	[33]
	*C. odorata* L.	Leaf essential oil	[32]
β-Santalene (**49**)	*C. fissilis* Vell.	Stem bark oil	[39]
Germacrene D (**50**)	*C. fissilis* Vell.	Leaf and stem bark oil	[39]
	*C. odorata* L.	Leaves	[38]
	*C. odorata* L.	Stem bark oil	[33]
Germacrene A (**51**)	*C. fissilis* Vell.	Leaf oil	[39]
	*C. odorata* L.	Leaves	[38]
	*C. odorata* L.	Leaf and stem bark oil	[33]
Bicyclogermacrene (**52**)	*C. fissilis* Vell.	Leaf and stem bark oil	[39]
		Leaves	[38]
	*C. odorata* L.	Leaves	[38]
β-Selinene (**53**)	*C. fissilis* Vell.	Leaf and stem bark oil	[39]
	*C. odorata* L.	leaves	[38]
β-Bisabolene (**54**)	*C. fissilis* Vell.	Leaf and stem bark oil	[39]
	*C. odorata* L.	Leaf essential oil	[32]
δ-Cadinene (**55**)	*C. fissilis* Vell.	Leaf and stem bark oil	[39]
	*C. odorata* L.	Leaves	[38]
	*C. odorata* L.	Leaf oil	[33]
	*C. odorata* L.	Leaf essential oil	[32]
Ledol (**56**)	*C. fissilis* Vell.	Stem bark oil	[39]
Spathulenol (**57**)	*C. fissilis* Vell.	Leaf and stem bark oil	[39]
	*C. odorata* L.	Leaves	[38]
	*C. odorata* L.	Leaf and steam bark oil	[33]
Caryophyllene oxide (**58**)	*C. fissilis* Vell.	Leaf oil	[39]
	*C. odorata* L.	Leaves	[38]
	*C. odorata* L.	Leaf oil	[33]
	*C. odorata* L.	Leaf essential oil	[32]
Globulol (**59**)	*C. fissilis* Vell.	Leaf oil	[39]
	*C. odorata* L.	Leaf oil	[33]
Viridiflorol (**60**)	*C. fissilis* Vell.	Leaf and stem bark oil	[39]
	*C. odorata* L.	Leaves	[38]
β-Eudesmol (**61**)	*C. fissilis* Vell.	Stem bark oil	[39]
α-Eudesmol (**62**)			
τ-Cadinol (**63**)			
τ-Muurulol (**64**)		Leaves and stem bark oil	[39]
α-Cadinol (**65**)		Stem bark oil	
(*E,E*)-Farnesol (**66**)			
α-Ylangene (**67**)	*C. odorata* L.	Leaves	[38]
β-Bourbonene (**68**)	*C. odorata* L.		
β-Cubebene (**69**)	*C. fissilis* Vell.		
β-Longipinene (**70**)	*C. fissilis* Vell.		
α-Guaiene (**71**)	*C. odorata* L.		
α-Himachalene (**72**)	*C. odorata* L.		
Seychellene (**73**)	*C. odorata* L.	Leaves	[38]
*cis-*4(14),5-Muuroladiene (**74**)	*C. odorata* L. *C. fissilis* Vell.		
β-Camigrene (**75**)	*C. odorata* L.		
γ-Himachalene (**76**)	*C. odorata* L.		
α-Selinene (**77**)	*C. odorata* L.		
	*C. odorata* L.	Leaf oil	[33]
Epizoraneno (**78**)	*C. fissilis* Vell.	Leaves	[38]
7-epi-α-Selinene (**79**)	*C. odorata* L.	Leaves	[38]
γ-Cadinene (**80**)	*C. odorata* L.	Leaves	[30]
		Leaves	[38]
	*C. odorata* L.	Steam bark oil	[33]
Germanecrene B (**81**)	*C. odorata* L.	Leaves	[38]
	*C. oaxacensis* C.DC. and Rose	Leaf oil	[32]
Elemol (**82**)	*C. odorata* L.	Leaves	[38]
	*C. odorata* L.	Steam bark oil	[33]
Humulene oxid (**83**)	*C. odorata* L.	Leaves	[38]
	*C. odorata* L.	Leaf essential oil	[32]
1-epi-Cubenol (**84**)	*C. odorata* L.	Leaves	[38]
epi-α-Muurolol (**85**)			
Selin-11-en-4-α-ol (**86**)			
Cedranodiol (**87**)			
Cembrene A (**88**)			
Cryptomeridiol (**89**)	*C. fissilis* Vell.	Roots	[27]
β-Funebrene (**90**)	*C. odorata* L.	Leaf essential oil	[32]
α-Santalene (**91**)			
*Z*-α-Bergamotene (**92**)			
epi-β-Santelene (**93**)			
Amorfa-4,11-dieno (**94**)			
β-Acoradieno (**95**)			
Isobicyclogermanecrene (**96**)			
*E*-β-bergamota (**97**)			
γ-Humulene (**98**)			
β-alaskene (**99**)			
Mintsulfide (**100**)			
14-oxi-α-muurolene (**101**)			
Isoledene (**102**)	*C. odorata* L.	Leaf and stem bark oil	[33]
Ylanga-2,4(15)-diene (**103**)		Leaf oil	
Pacifigorgia-2,10-diene (**104**)		Leaf oil	
Sesquisabinene (**105**)		Stem bark oil	
(*E*)-β-Farnesene (**106**)		Stem bark oil	
allo-aromadendrene (**107**)		Stem bark oil	
ar-curcumene (**108**)		Stem bark oil	
Zingiberene (**109**)		Stem bark oil	
β-sesquiphellandrene (**110**)		Stem bark oil	
Cadina-1,4-diene (**111**)		Leaf oil	
α-Calacorene (**112**)		Leaf oil	
(*E*)-Nerolidol (**113**)		Leaf oil	
Alismol (**114**)		Stem bark oil	
ar-turmerone (**115**)		Stem bark oil	
(*E*)-α-atlantone (**116**)		Stem bark oil	
7-hydroxycalamenene (**117**)	*C. odorata* L. grafted with *Toona australis* (F. Muell.) Harms	Stems	[24]
5-hydroxycalamenene (**118**)			
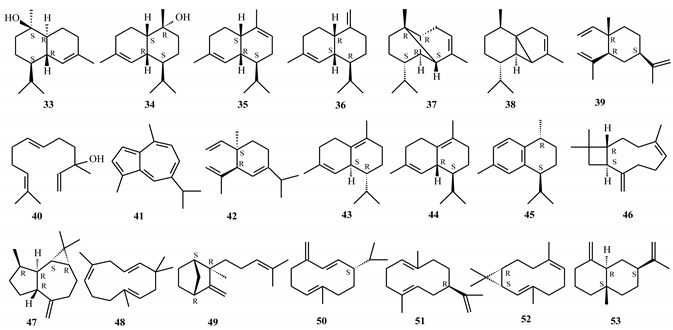			
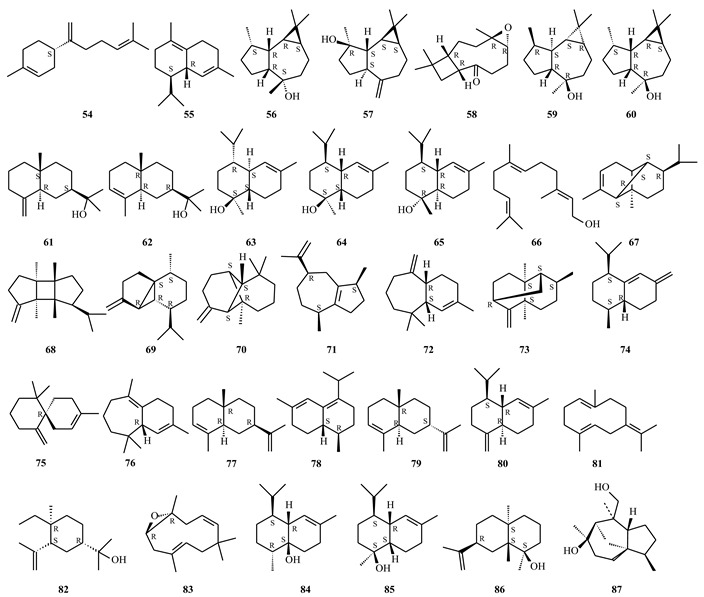			
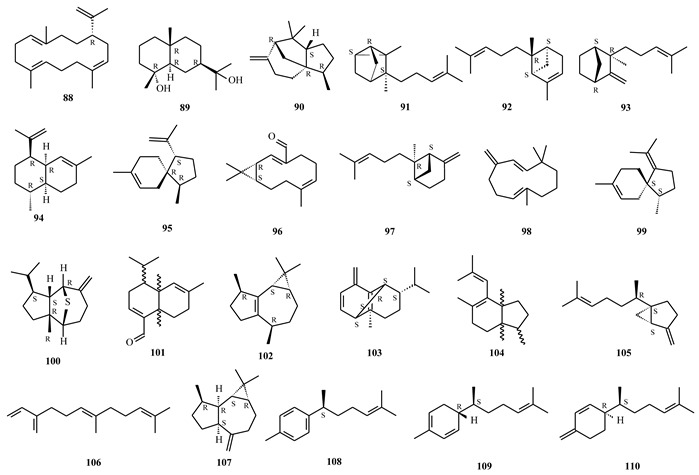			
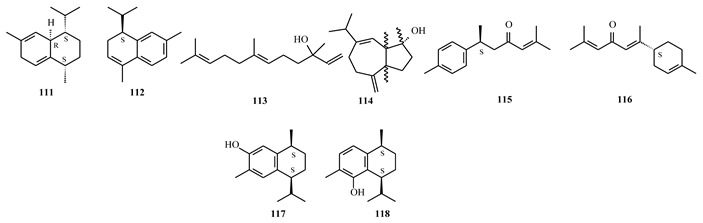			

**Table 6:** Triterpenes isolated from the genus *Cedrela* (1960–2020).

Compound	Plant Species	Plant Part	References
3,11-dioxoolean-12-en-28-onic acid (**119**)	*C. fissilis* Vell.	Fruits	[27]
Ursolic acid (**120**)	*C. odorata* L.	Stems	[28]
Oleanolic acid (**121**)	*C. fissilis* Vell.	Roots	[43]
		Fruits	[27]
	*C. montana* Moritz ex Turcz.	Fruits and seeds	[44]
	*C. odorata* L.	Stems	[28]
Oleanonic acid (**122**)	*C. odorata* L.	Bark	[21]
	*C. fissilis* Vell.	Roots	[27]
		Fruits	[27]
	*C. montana* Moritz ex Turcz.	Fruits and seeds	[44]
3-oxo-11α,12α-epoxi-olean-28,13β-olide (**123**)	*C. montana* Moritz ex Turcz.	Fruits and seeds	[44]
3-oxo-olean-11-en-28,13β-olide (**124**)		Fruits and seeds	
Cedrelosin C (**125**)	*C. odorata* L.	Leaves	[45]
Cedrelosin D (**126**)		Leaves	
Odoratol (**127**)	*C. odorata* L. grafted with *Toona australis* (F. Muell.) Harms	Stems	[24]
	*C. odorata* L.	-	[46]
		-	[47]
		Heartwood	[48]
	*C. odorata* L.	-	[49]
	*C. odorata* L.	Heartwood	[50]
	*C. fissilis* Vell.	Stems	[27]
Odoratone (**128**)	*C. odorata* L.	-	[46]
		-	[47]
	*C. odorata* L.	Heartwood	[50]
	*C. fissilis* Vell.	Stems	[27]
	*C. odorata* L. grafted with *Toona australis* (F. Muell.) Harms	Stems	[24]
Iso-odoratol (**129**)	*C. odorata* L.	-	[47]
	*C. odorata* L.	Heartwood	[50]
	*C. fissilis* Vell.	Stems	[27]
	*C. odorata* L. grafted with *Toona australis* (F. Muell.) Harms	Stems	[24]
22S,3α-dihydrotirucala-7,24-dien-23-one (**130**)	*C. fissilis* Vell.	Stems	[27]
Pentaol (**131**)	*C. odorata* L.	Wood	[51]
	*C. fissilis* Vell.	Stems	[27]
	*C. odorata* L. grafted with *Toona australis* (F. Muell.) Harms	Stems	[24]
Piscidinal A (**132**)	*C. fissilis* Vell.	Stems	[27]
	*C. odorata* L.	Twigs and leaves	[52]
Hispidol A (**133**)	*C. fissilis* Vell.	Stems	[27]
	*C. odorata* L.	Twigs and leaves	[52]
25-methoxyhispidol A (**134**)	*C. odorata* L.	Twigs and leaves	[52]
Sapelin F (**135**)			
Cedrodorol A (**136**)			
Cedrodorol B (**137**)			
3-oxo-threo-23,24,25-trihydroxytirucall-7-ene (**138**)	*C. odorata* L.	Heartwood	[21]
24-methylene-26-hydroxycycloartan-3-one (**139**)	*C. fissilis* Vell.	Roots	[27]
Nilocetina (**140**)			
Sapelin B (**141**)	*C. odorata* L.	Twigs and leaves	[52]
21α-metilmelianodiol (**142**)			
Altissimanin A (**143**)			
Cycloartenol (**144**)	*C. odorata* L. grafted with *Toona australis* (F. Muell.) Harms	Stems	[24]
24-methylenecycloartan-3β-ol (**145**)			
Cycloartenone (**146**)			
24- methylenecycloartan-3-one (**147**)			
Tirucallol (**148**)	*C. odorata* L. grafted with *Toona australis* (F. Muell.) Harms	Stems	[24]
22ξ-hydroxytirucalla-7,24-dien-3,23-dione (**149**)			
22ξ,3α-hydroxytirucalla-7,24-dien-23-one (**150**)			
22ξ,3β-hydroxytirucalla-7,24-dien-23-one (**151**)			
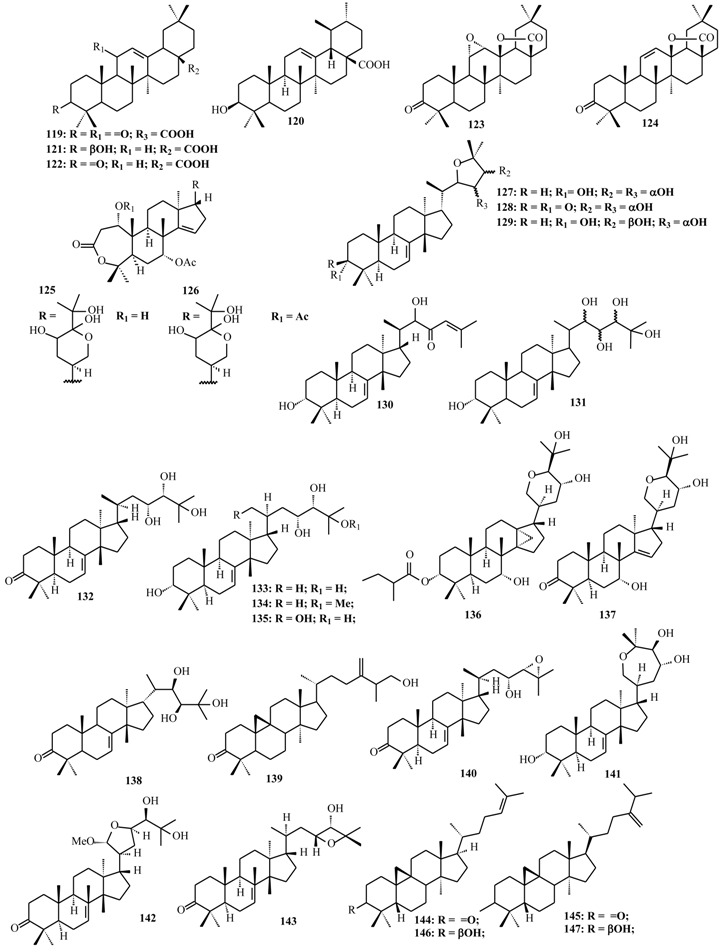			
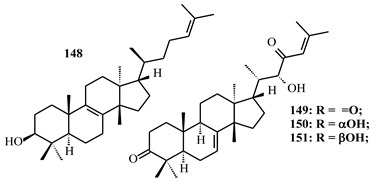			

**Table 7:** Cycloartanes isolated from the genus *Cedrela* (1960–2020).

Compound	Plant Species	Plant Part	References
Cycloeucalenol (**152**)	*C. odorata* L. grafted with *Toona australis* (F. Muell.) Harms	Stems	[23,24]
3β-*O*-β-d-glucopyranosylcycloeucalenol (**153**)	*C. odorata* L. grafted with *Toona australis* (F. Muell.) Harms	Stems	[23,24]
3β-*O*-(2′,3′,4′,6′-Tetra-*O*-acetil-β-d-glucopyranosyl) Cicloeucalenol (**154**)			[23]
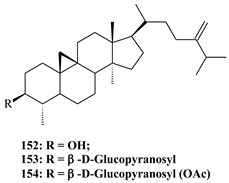			

**Table 8:** Steroids isolated from the genus *Cedrela* (1960–2020).

Compound	Species	Plant Part	References
2α,3α,20-trihydroxy-16β-acetoxy-20(R) pregnane (**155**)	*C. odorata* L.	Leaves	[45]
2β,3β-Dihydroxypregnan-16-one (**156**)			
3β-*O*-β-d-glucopyranosyl-24-methylenocolesterol (**157**)	*C. odorata* L.	Heartwood	[21]
β-sitosterol (**158**)	*C. odorata* L.	Bark and sapwood essential oil	[21]
	*C. odorata* L.	Leaves	[30]
	*C. odorata* L. grafted with *Toona australis* (F. Muell.) Harms	Stems	[23]
	*C. fissilis* Vell.	-	[27]
	*C. montana* Moritz ex Turcz.	Fruits and seeds	[44]
Stigmaterol (**159**)	*C. odorata* L. grafted with *Toona australis* (F. Muell.) Harms	Stems	[23]
	*C. fissilis* Vell.	-	[27]
	*C. montana* Moritz ex Turcz.	Fruits and seeds	[44]
Campesterol (**160**)	*C. odorata* L. grafted with *Toona australis* (F. Muell.) Harms	Stems	[23]
	*C. fissilis* Vell.	-	[27]
3β-*O*-β-d-glucopyranosylsitosterol (**161**)	*C. fissilis* Vell.	-	[27]
Sitostenone (**162**)		-	
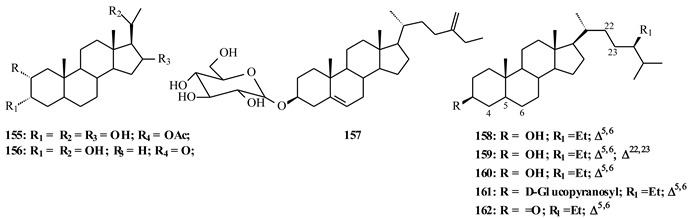			

**Table 9:** Limonoids isolated from the genus *Cedrela* (1960–2020).

Compound	Plant Species	Plant Part	References
Odoratina (**163**)	*C. odorata* L.	Heartwood	[48]
Cedrelanolideo I (**164**)	*C*. *salvadorensis* Standl.	Stem bark	[59,60,61]
	*C. dugesii* S.Watson *C*. *salvadorensis* Standl.	Heartwood	[62]
(3β,13α,14β,15β,17α)-14,15:21,23-diepoxi-3,6-dihydroxy-4,4,8-trimetil-24-norchola-11,20,22-trien-7-ona (**165**)	*C. odorata* L.	Leaves	[63]
Azadiradione (**166**)	*C. odorata* L. grafted with *Toona australis* (F. Muell.) Harms	Stems	[23]
		Stems	[24]
20,21,22,23-tetrahydro-23-oxoazadiradione (**167**)	*C. odorata* L. grafted with *Toona australis* (F. Muell.) Harms	Stems	[23]
		Stems	[24]
	*C. fissilis* Vell.	Leaves	[27]
Kihadalactone A (**168**)	*C. odorata* L.	Leaves	[45]
7α-11β–diacetoxydihydronomilina (**169**)	*C. odorata* L.	Fruits	[64]
	*C. odorata* L.	Leaves	[63]
11β-acetoxyobacunil acetate (**170**)	*C. odorata* L.	Stem bark	[65]
11β-acetoxyobacunol (**171**)		Stem bark	
11β, 19-diacetoxy-1-deacetil-1-Epidihydronomilina (**172**)		Stem bark	
7-acetyldihydronomilin (**173**)	*C. odorata* L.	Stem bark	[65]
	*C. odorata* L.	Leaves	[45]
7-acetil-11β-acetoxydihydronomilina (**174**)	*C. odorata* L.	Stem bark	[65]
Odoralide (**175**)			
Delevoyin D (**176**)	*C. odorata* L.	Leaves	[45]
(23*R*)-photogedunina (**177**)	*C. dugesii* S.Watson	Heartwood	[65]
	*C. dugesii* S.Watson *C*. *salvadorensis* Standl.	Heartwood	[66]
(23*S*)-photogedunina (**178**)	*C. dugesii* S.Watson	Heartwood	[62]
	*C. dugesii* S.Watson *C*. *salvadorensis* Standl.	Heartwood	[66]
7-deacetylgedunin (**179**)	*C. odorata* L. grafted with *Toona australis* (F. Muell.) Harms	Stems	[23]
		Stems	[24]
	*C. fissilis* Vell.	Leaves	[67]
7-desacetoxy-7-oxogedunin (**180**)	*C. odorata* L.	-	[68]
		Heartwood	[69]
	*C. odorata* L. grafted with *Toona australis* (F. Muell.) Harms	Stems	[23]
		Stems	[24]
	*C. fissilis* Vell.	Leaves	[27]
		Leaves	[67]
Gedunin (**181**)	*C. odorata* L.	Heartwood	[69]
		Sapwood	[21]
	*C. odorata* L.	Wood	[70]
		-	[71,72]
	*C. odorata* L. grafted with *Toona australis* (F. Muell.) Harms	Stems	[23]
		Stems	[24]
	*C. fissilis* Vell.	Roots	[67]
		Roots	[27]
	*C. salvadorensis* Standl.	Bark	[73]
Photogedunin (**182**)	*C. odorata* L.	Heartwood	[74]
	*C. fissilis* Vell.	Leaves	[67]
		Leaves	[27]
	*C. salvadorensis* Standl.	Heartwood	[75]
	*C. montana* Moritz ex Turcz.	Fruits and seeds	[44]
	*C. dugesii* S. Watson	Heartwood	[66]
	*C. odorata* L. grafted with *Toona australis* (F. Muell.) Harms	Stems	[23]
		Stems	[24]
1α-methoxy-1,2-dihydrogedunin (**183**)	*C. odorata* L. grafted with *Toona australis* (F. Muell.) Harms	Stems	[24]
		Stems	[24]
6α-acetoxygedunina (**184**)	*C. fissilis* Vell.	Roots	[67]
1,2-di-hydro-3 β-hydroxy-7-desacetoxy-7-oxogedunina (**185**)	*C. fissilis* Vell.	Leaves	[67]
Toosendanin (**186**)	*C. dugesii* S.Watson *C*. *salvadorensis* Standl.	Heartwood	[62]
Febrifugin (**187**)	*C. odorata* L. grafted with *Toona australis* (F. Muell.) Harms	Stems	[24]
		Stems	[23]
3β-deacetylfissinolide (**188**)		Stems	[23]
		Stems	[24]
3β– Hydroxyisomexicanolideo (**189**)	*C. fissilis* Vell.	Seeds	[76]
Angustinolide (**190**)	*C. angustifolia* DC.	Seeds	[77]
Angustidienolide (**191**)		Seeds	
2α-Hydroxyangustidienolide (**192**)		Seeds	
6-hydroxycarapin (**193**)	*C. odorata* L.	Wood	[51]
Mexicanolide (**194**)	*C. odorata* L.	Wood	[78]
		Wood	[79]
		Wood	[80]
		Wood	[81]
		Seeds	[82]
	*C. fissilis* Vell.	Seeds	[83]
	*C. odorata* L.	Heartwood	[84]
Fissinolide (**195**)	*C. fissilis* Vell.	Fruits	[76]
6–desoxiswietenolide (**196**)	*C. odorata* L.	Seeds	[82]
6α-hydroxymexicanolide (**197**)			
Cedrelosin E (**198**)	*C. odorata* L.	Leaves	[45]
Cedrodorin (**199**)	*C. odorata* L.	Stem bark	[65]
		Leaves	[85]
6-acetoxycedrodorin (**200**)	*C. odorata* L.	Leaves	[85]
6-desoxi-9α-Hydroxycedrodorina (**201**)		Leaves	
9α-hydroxycedrodorina (**202**)		Leaves	
8β, 14α-dihydroswietenolide (**203**)	*C. odorata* L.	Stem bark	[65]
3β,6-dihydroxydihydrocarapina (**204**)		Stem bark	[65]
		Leaves	[45]
3β-hydroxydihydrocarapin (**205**)		Stem bark	[65]
Swietenolide (**206**)		Stem bark	[65]
		Leaves	[45]
Xiloccensin K (**207**)	*C. odorata* L.	Stem bark	[65]
3β-acetoxycarapin (**208**)	*C. fissilis* Vell.	Roots	[43]
		Roots	[27]
Andirobin (**209**)	*C. odorata* L.	Seeds	[82]
Methylangolensate (**210**)	*C. odorata* L.	Heartwood	[69]
		-	[47]
	*C. odorata* L. grafted with *Toona australis* (F. Muell.) Harms	Stems	[23]
		Stems	[24]
	*C. fissilis* Vell.	Leaves	[27]
Cedrelosin A (**212**)	*C. odorata* L.	Leaves	[45]
11β,7α-limonol-diacetate (**212**)		Leaves	
7α-limonylacetate (**213**)		Leaves	
Limonol (**214**)		Leaves	
Cedrelosin B (**215**)		Leaves	
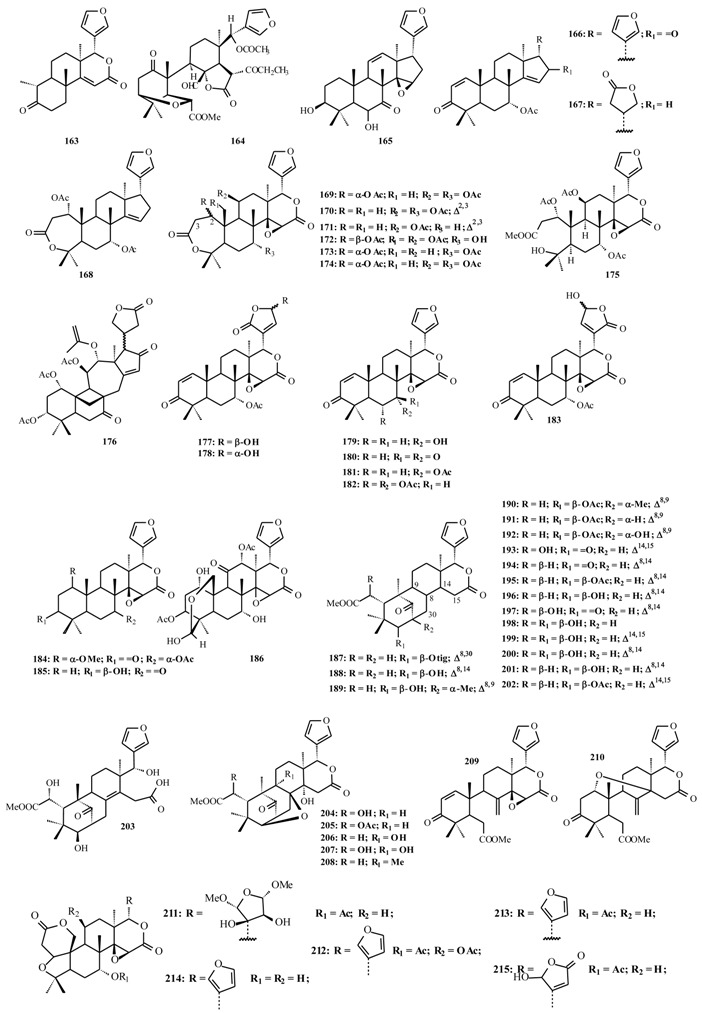			

**Table 10:** Analysis of limonoid classes and types from *Cedrela* conforming to Fang et al. [16] classifications.

Class	Type	Ring				Side-Chain	Limonoids	%	
		A	B	C	D			Type	Class
D-seco	Gedunin	i	i	i	iii	ii	**177**–**185**	17.0	17.0
Intact Rings	Delevoyin	i	i	iv	i	iii	**176**	1.9	9.4
	Havenensin	i	i	i	i	ii	**165**–**167** and **186**	7.5	
B,D-seco	Andirobin	i	v	i	iii	ii	**164**, **209** and **210**	5.7	47.2
	Mexicanolide	i	vi	i	iii	ii	**187**–**208**	41.5	
A-seco	Neotecleanin	vi	i	i	i	ii	**211**–**215**	9.4	11.3
	Evoludone	iii	i	i	i	ii	**168**	1.9	
A,D-seco	Obacunol	iii	i	i	iii	ii	**169**–**175**	13.2	13.2
-	Degraded *	-	-	i	iii	ii	**163**	1.9	1.9

* Fang et al. [15] did not relate degraded limonoids to any class. i—intact. ii—furan. iii—lactone. iv—expanded. v—open. vi—recyclized.

**Table 11:** The half maximal inhibitory concentration (IC_50_) values for *Cedrela* active species extracts obtained in the table presented by MacKinnon et al. [70].

Species	Part Organ	Collection Site	IC_50_ (µg/mL) Clone D6	IC_50_ (µg/mL) Clone W2
*C. salvadorensis*	Bark	Costa Rica	>20	15.0
	Fruits		18.7	15.3
	Leaves		8.30	>20
	Wood		13.8	13.2
*C. odorata*	Wood	Costa Rica	1.37	1.25
	Wood	Belize	1.20	1.11
	Wood	Togo	9.29	2.77
